# Cultural factors that influence the treatment of osteosarcoma in Zulu patients: Healthcare professionals’ perspectives and strategies

**DOI:** 10.4102/hsag.v23i0.1095

**Published:** 2018-06-28

**Authors:** Ottilia Brown, Veonna Goliath, Dalena R.M. van Rooyen, Colleen Aldous, Leonard C. Marais

**Affiliations:** 1 School of Clinical Medicine, University of KwaZulu-Natal, South Africa; 2 Department of Social Development Professions, Nelson Mandela Metropolitan University, South Africa; 3 Faculty of Health Sciences, Nelson Mandela Metropolitan University, South Africa

## Abstract

**Purpose:**

International and national research regarding the discussion of cancer treatment across cultural boundaries is sparse. This study was conducted in the province of KwaZulu-Natal, South Africa, where healthcare encounters are largely culturally discordant; and this study focused on adult Zulu patients diagnosed with osteosarcoma. The purpose of this research study was to identify the cultural factors associated with discussing the different treatment options – and to explore healthcare professionals’ responses to these cultural factors – from the healthcare professionals’ perspective.

**Methods:**

A qualitative, exploratory, descriptive and contextual research design was used. We conducted focus group interviews with professional nurses, allied health professionals and orthopaedic physicians. These three focus groups comprised a total of 23 participants, and interviews were conducted with each of these groups. We thematically analysed the interview transcripts, using Guba’s model of trustworthiness to ensure rigour.

**Results:**

We found that the factors, influencing treatment discussions in this cross-cultural clinical setting, included the meaning and the disclosure of cultural health beliefs.

We identified strategies for responding to the cultural factors associated with amputation, namely timing treatment discussions, using support services, patient models and DVDs or videos. Strategies for responding to cultural and health beliefs that affect the treatment included initiating the cultural discussion, demonstrating an understanding of patients’ cultural beliefs and liaising with family and cultural decision-makers wherever possible.

**Conclusion:**

Our findings emphasised healthcare professionals’ reports of how patients can experience the discussion of culturally discordant treatment options as bad news. We recommend that the treatment discussion form an integral part of the guidelines for culturally competent communication with such cancer patients.

## Introduction

An extensive literature exists on communicating the diagnosis and prognosis of cancer to patients (Figg et al. 2010; Girgis & Sanson-Fisher 2010; Hagerty et al. 2005; Monden, Gentry & Cox 2016). However, the literature on discussing treatment options tends to be limited (Baile et al. 2000; Girgis & Sanson-Fisher 2010). Research, regarding the discussion of treatment options across cultural boundaries, is especially sparse. Although informing patients of their treatment options and ensuring their understanding in this regard is considered good practice (Girgis & Sanson-Fisher 2010). Patients tend to be less satisfied with discussions regarding treatments when compared with those in which the diagnosis only is communicated (Galletari et al. 2002).

This study was conducted at a public tertiary hospital in a unit dedicated to the treatment of musculoskeletal tumours in the predominantly rural province of KwaZulu-Natal, South Africa. At this facility, healthcare encounters are largely culturally discordant. The study focused on adult Zulu patients diagnosed with osteosarcoma. Osteosarcoma is the most common primary bone cancer (Ritter & Bielack 2010) and the complexity of its treatment necessitates a multidisciplinary team (MDT), approach.

This context and the disease type were chosen, because previous research at our study site reported that the majority of patients present with locally advanced or metastatic disease (Ferreira & Marais 2012). This limits the treatment options and it usually results in a very poor prognosis (Errani et al. 2011; Ferreira & Marais 2012; Marais et al. 2015; Meazza & Scanagatta 2016; Ritter & Bielack 2010).

Although there has been a significant shift with regard to surgical treatment options, away from amputation to limb salvaging (around 80% of patients) (Bielack et al. 2009; Jaffe 2009), a substantial proportion of patients, presenting at the study site, are not candidates for limb salvaging because of the advanced stage of the disease at presentation (Ferreira & Marais 2012). Granted that this late presentation is partly a result of misdiagnosis at community health centres or district hospitals (Ferreira & Marais 2012), nevertheless observations in clinical practices indicate that Zulu patients often prefer to exhaust all the traditional healing options before seeking Western medical assistance.

Zulu patients typically engage in cultural health beliefs and practices that are in contrast to the Western medical model, within which medical training is housed. They may conceive illness as resulting from displeasing the ancestors, witchcraft or troublesome social relationships (Mdondolo, De Villiers & Ehlers 2003; Vorobiof, Sitas & Vorobiof 2001). The purpose of this research study therefore was to identify the cultural factors associated with discussing treatment options – and to explore the healthcare professionals’ (HCPs) responses to these cultural factors – from the HCPs’ perspective. This research forms part of a larger research project and, although this article focuses on HCPs’ perspectives, patient interviews were also conducted and will be reported on in a separate publication.

## Research methodology

### Research design and methods

We used a qualitative, exploratory, descriptive and contextual approach, and we conducted focus group interviews with three distinct groups of professionals: professional nurses, allied health professionals and orthopaedic physicians (including consultants and registrars). The focus group interview schedule was piloted with a social-work colleague who has experience with working with cancer patients in the study setting. The interview questions focused on approaches that HCPs take when discussing the treatment of osteosarcoma with Zulu patients and the cultural considerations pertaining to this discussion as well as their responses to these cultural factors.

### Participants and sampling

We recruited the participants, using census sampling. All 23 study participants were members of the MDT involved with the care of Zulu patients with osteosarcoma. The MDT at the study site comprised orthopaedic consultants and registrars, professional nurses from the orthopaedic and oncology outpatient clinics and wards, allied health professionals including dieticians, occupational therapists, physiotherapists, social workers and clinical psychologists. Five team members could not participate because of scheduling conflicts. Only four of the participants were isiZulu speaking, highlighting the culturally discordant medical encounters at this health facility. For the purposes of describing the sample and contextualising the findings, the demographic details of the sample are outlined in Table 1.

**TABLE 1:** Focus group demographic information (*n* = 23).

Demographic information	Sample size (*n*)
Orthopaedic consultants and registrars	9
Registered nurses from orthopaedic wards, orthopaedic clinic, oncology clinic and pain service	5
Allied health professionals (physiotherapists, occupational therapists, dieticians, social worker)	9
**Gender**	
Males	8
Females	15
**Ethnic groups in South Africa**	
White people	13
Indian people	4
African people (isiZulu)	4
Mixed race people	2

### Data collection and analysis

The second author, an experienced qualitative interviewer, conducted the focus groups, because she had no prior knowledge of the participants. The focus group interviews were conducted at the hospital, as the participants work in a resource-constrained environment, and the interviews ranged in duration from 54 to 95 min. Because of scheduling conflicts, the distinctive groups of professionals (orthopaedic physicians, nurses and allied health professionals) were interviewed separately. All the interviews were audiotaped, transcribed verbatim and analysed for themes (Braun & Clarke 2006). The data were independently coded by the focus group interviewer and the primary investigator. They were further reviewed by two qualitative research experts before consensus was reached on the themes.

### Trustworthiness

Guba’s model of trustworthiness (Lincoln & Guba 1985) was used to ensure rigour. With regard to the criterion of credibility, research methods, which are well-established in the qualitative genre, were used. The researchers were suitably qualified, and they had the relevant experience required for the research project. Data verification entailed the processes of independent coding by the second author, two rounds of peer review of the themes by two independent qualitative researchers, and a final theme discussion by the principal investigator and the second author.

Contextual credibility was addressed, because the principal investigator had in-depth knowledge of the participating organisation. This ensured that the focus group interviewer had a good understanding of the approach taken in the management of osteosarcoma patients at the study site. The focus group interviewer employed iterative questioning and probing to elicit rich data and verify the information.

Guba’s transferability and dependability were addressed by providing a detailed description of the research context and the research procedures followed so that readers would be able to decide on the transferability of the findings for their context – and also to facilitate any future repetition of the research project. Dependability was further addressed by providing an operational description of the research design and methods as well as a reflexive appraisal of the research undertaken.

The confirmability criterion relates to ensuring that the findings reflected the experiences and opinions of the participants, rather than those of the researcher (Shenton 2004). As the principal investigator works at the study site, an experienced independent researcher, who had had no prior contact with the participants, conducted the focus groups. The process of bracketing was used while analysing the data (Tufford & Newman 2010), and the themes were verified by independent qualitative researchers.

### Ethical approval

Anonymity was ensured, as the focus group interviewer had not had any prior contact with the participants. The purpose, objectives and significance of the study were explained to the participants before consent was obtained and these aspects were also outlined in written form. Voluntary participation, the right to withdraw from the study, confidentiality and informed consent therefore were applied.

Ethical clearance to conduct the study was obtained from the Biomedical Research Ethics Committee at the University of KwaZulu-Natal. Consent was obtained from Health Research and Knowledge Management of the Department of Health KwaZulu-Natal, following which gatekeeper consent was obtained from the CEO of the tertiary hospital where the research was conducted.

## Results

The results highlight two significant themes pertaining to the focus of the article. The first theme addressed the cultural factors associated with the treatment discussion, while the second theme focused on the strategies used to respond to these factors. Importantly, the focus group process was transformative (De Laine 2000), as it allowed for the Zulu HCPs to educate other participants about Zulu cultural beliefs and practices. This enhanced the awareness of cultural factors to be considered when communicating with Zulu patients.

### Cultural factors influencing the treatment of osteosarcoma

The participants reported that culture plays a significant role in the discussion of treatment.

#### Zulu cultural and health beliefs

The HCPs indicated that some patients were concerned about community exclusion post-amputation. They also reported that Zulu patients’ cultural beliefs dictate that they cannot become an ancestor if they have had an amputation, because their body would subsequently be incomplete. The participants suggested that these cultural beliefs were changing, as more patients were consenting to amputation. They attributed this change in belief to education initiatives on the radio:

‘… [*T*]hat’s part of the belief … once you have an amputation then you don’t really belong to that community … they don’t really accept you that well …’

‘… [*A*]nd he died from it in the end, from a giant cell tumour … he said if you amputate me I won’t become an ancestor, so I am refusing …’

‘Is that changing? Because we are getting more consent for amputations …’

It is changing; there are a lot of discussions that are actually going on about that … Radio works well, they speak about these things [*cultural beliefs*].’

The participants indicated that the patients or their elders wanted to engage in traditional healing – to achieve a cure and prevent the need for amputation. Traditional healers can claim cures, which result in the community rejoicing. Patients preferred to go home, as opposed to complying with HCPs’ proposal to invite family members to the hospital. The participants were concerned that the patients would neglect Western medicine, when traditional options were seen by the patients as mandatory:

‘… [*T*]hey will tell you; I want to go and see a traditional healer.’

‘So the intention with going to consult a traditional healer is because you, as a patient, believe that there is [*a*] cure …’

‘… I do try and see if we can get any means to get the family to come; but most of the time they want to go back home rather than bringing someone.’

‘So how about going back home … they (the elders) will point out there is a good traditional healer that might help … but now they forget about the Western part of medicine.’

The Zulu HCPs discussed the cultural hierarchies in Zulu families and the significance of these hierarchies in decision-making. The participants reported that the patients need to consult with their elders before agreeing to treatment, even when they understand the nature of the condition:

‘… [*W*]e have hierarchies at home, from my father, to go down to my uncles and everybody … And then the issue will be discussed with all of them and then they will come up with their own inputs …’

The HCPs were concerned that the cultural decision-maker might not have insight into the patient’s condition and that such patients would be deserted by the cultural hierarchy if they ignored their advice and made decisions independently.

‘If you disregard or ignore her decisions, she [*an identified elder*] will decide to pull out and you feel that you are on your own … If let’s say you have made a bad decision, she will say, but I told you and you went on and you got it wrong and you listened to those people, now you are on your own …’

#### Patient disclosure of traditional beliefs to providers

The HCPs perceived that patients’ willingness to share their cultural beliefs was an individual preference. Some patients withheld their desire to consult a traditional healer, while others admitted this need. There was no consensus regarding patients’ disclosure preferences. Some of the patients disclosed their traditional beliefs with greater ease to Zulu HCPs, whereas others more easily disclosed these beliefs to non-Zulu caretakers when prompted.

‘You find that there’s a sister at our front desk, let’s say it’s a white sister or an Indian sister; she will prefer to come to me maybe because of the language barrier.’ (Zulu nurse participant)

‘Most of the time, if you actually speak to them [*Zulu patients*], [*and ask them*] are you going to go and see someone [*traditional healer*], they will tell you, well actually … you see …’

Conversely, the participants observed a link between the patients’ reluctance, fear or embarrassment to disclose cultural beliefs and their perception that Western medicine was contrary to their cultural beliefs. Such patients may therefore perceive that HCPs would try to prevent their compliance with cultural expectations:

‘I think they are scared to tell us because they think we believe that it’s, you know … And that we are going to try and stop them.’

‘Especially that they know that at the hospital there’s that thing of medical, you know, against cultural …’

### Responding to cultural factors associated with the treatment discussion

Strategies for responding to the cultural factors associated with amputation and patients’ cultural and health beliefs emerged from the data.

#### Strategies for responding to the cultural factors associated with amputation

Timing the treatment discussion was viewed as a means of preventing the patient from signing a refusal of hospital treatment before diagnostic testing was complete. The HCPs emphasised the importance of not answering any questions regarding treatment options for osteosarcoma before the diagnosis had been confirmed.

‘I generally then stop and say, well, let’s not talk about that now; let’s focus on finding out what it is first, and then once we know we will talk about what the possibilities are for treatment after that.’

Another proposed strategy involved introducing the patients to veteran patients. The participants suggested accessing patients who had been through the process successfully as models, including a patient model in the MDT to inspire newly diagnosed patients and making a video of patients with successful outcomes. Exposure to veteran patients could result in ongoing support and demonstrate survival and the efficacy of Western medicine. However, the possibility that such patient models could die should be considered when making these introductions. Using known characters or celebrities with access to better resources could create false expectations and this should therefore be avoided:

‘Like somebody who has gone through it (Zulu patient with amputation) and they can say, look I am out of it on the other side; this has actually helped me.’

The participants tended to respond to the refusal of amputation by (1) offering the patients other treatment options like chemotherapy; (2) facilitating follow-up with oncology and other services like psychology, social work and dietetics; and (3) mobilising support by including the psychologist in diagnosis and treatment discussions wherever possible.

‘… [*B*]ecause if we get a diagnosis and he then refuses amputation but is willing to stay we can still do … chemo and (facilitate follow-up from) dietitians and psychologists … oncology.’

#### Strategies for responding to cultural and health beliefs that affect treatment

The participants reported needing to balance cultural sensitivity with the urgency for prompt treatment. HCPs accepted their onus to initiate discussions on cultural requirements in order to fast-track the decision-making process.

‘… [*A*]sk this patient what is important to them and how do they see themselves managing this, do they feel they need to go to the Sangoma (traditional healer). I think time is also wasted because we expect the patient to tell us their needs, like … I want to go home to discuss this … whereas if we initiated it and said, what do you want to do now, what does your family think needs to happen, we would maybe know on day one or day two …’

The HCPs emphasised the importance of acknowledging patients’ need to discuss treatment with their families and encouraging patients to engage in their cultural traditions. They suggested that patients’ guilt regarding choosing Western medicine could be mediated by encouraging cultural rituals. Those participants also reported encouraging patients to follow Western and traditional approaches for managing health and illness:

‘… I do understand that you, you want to discuss with the family …’

‘… [*Y*]ou don’t want them to feel guilty about not following the culture but following the Western medicine culture.’

The participants reported that they tried to liaise directly with the family and cultural decision-makers, wherever possible. This practice improved communication. Negotiation was frequently used to persuade patients not to go home, but rather suggesting they invite family members to the hospital:

‘… [*I*]f they say they want to speak to their family, what I usually do [*is ask the patient*], can’t you get someone to come so that we can explain to them and then that person can go and explain to the family what is happening …’

The HCPs reported that when patients insisted on going home, they then gave the patient a deadline for returning from the family consultation. They explained to the patient that the traditional healer would not be able to assist – just as Western medicine could not cure every illness. They also reiterated with patients that the final decision was with them and not with the family. The participants ensured that patients were always well-informed:

‘… I do explain to them that, okay, it is your decision, whatever treatment options that we are going to give you. It’s your decision to make; it’s not somebody else’s decision at home …’

The participants proposed specific strategies for culturally competent communication. They reiterated not making assumptions based on culture and race, but they proposed taking responsibility for learning about the Zulu culture and suggested including traditional healers in the regimen for patient care.

‘I think culture is very specific to an individual; we mustn’t think that all black patients are going to have that same culture …’

‘… [*T*]he Department of Health as a government department needs to find a way to incorporate traditional healers into the medical setting.’

## Discussion

The HCPs in this study demonstrated considerable knowledge of Zulu cultural beliefs and practices - a characteristic deemed essential for working in cross-cultural clinical settings (Mullin, Cooper & Eremenco 1998; Pierce 1997; Tucker et al. 2013). They emphasised taking responsibility for learning to know the Zulu culture (Matthews-Juarez & Juarez 2011; Muñoz-Antonia 2014; Pesquera, Yoder & Lynk 2008). Cultural aspects reported on included the belief of having to remain intact to become an ancestor after death, the fear of being excluded from the community post-amputation, the belief in traditional healing as a means of cure, and the role of the cultural hierarchy in decision-making. The HCPs also reflected on patients’ disclosure preferences and patients’ hesitation to disclose their cultural beliefs – given their perception that Western HCPs may obstruct their desire to observe their traditions (Davis, Oh, Butow, Mullan & Clarke 2012; Robinson & McGrail 2004; Shelley et al. 2009).

The HCPs reported on patients’ cultural beliefs regarding amputation. Proposed strategies for responding to Zulu patients’ responses to amputation included timing the treatment discussion and using patient models – or videos of patients who have successfully rehabilitated after amputation – as a means of easing patients’ anxieties and facilitating decision-making (Baile & Beale 2001; Krouse 2001; Schofield et al. 2008). The participants ensured that the patients received care – whether or not they chose amputation. If the patients refused amputation, the HCPs offered other treatment options, facilitated follow-up from oncology and allied health professionals and mobilised support by including the psychologist in diagnosis and treatment discussions where possible (Baile & Beale 2001; Girgis & Sanson-Fisher 2010).

The participants reported on patients’ belief in traditional healing. Strategies for responding to this cultural factor included encouraging patients to engage in their traditions, demonstrating respect for patients’ preference to consult traditional healers and proposing that patients combine traditional and Western medicine (Broome & Broome 2007). Those patients with traditional belief systems associate consulting a traditional healer with hoping for a cure and receiving spiritual and physical profit from the consultation (Muhamad, Merriam & Suhami 2012). The participants were concerned about the paradigm divide on the hope of a miracle cure from traditional healers versus the Western medical message of no cure (Summerton 2006).

Research has shown that patients from indigenous populations, especially when diagnosed with a life-threatening illness such as cancer, may integrate Western medicine and traditional healing (Broome & Broome 2007; Muhamad et al. 2012; Struthers & Eschiti 2004). However, our study highlighted a concern that patients might neglect Western medicine when they returned home to engage in their traditions. It was also the HCPs’ experience that patients tended to delay returning to the hospital or avoided medical treatment completely.

Proposed strategies for fast-tracking treatment decision-making included initiating cultural discussions, liaising directly with the cultural decision-makers regarding treatment (Barclay, Blackhall & Tulsky 2007; Broome & Broome 2007) and suggesting that family members come to the hospital rather than the patients going home. Most patients, using alternatives to Western medicine, may have had an expectation of the clinician to initiate the discussion regarding these practices (Shelley et al. 2009). Furthermore, given the fact that the Zulu culture is located in a collectivistic paradigm where patients tend to defer to the collective for decision-making (Iwelunmor, Newsome & Airhihenbuwa 2014), liaising directly with cultural decision-makers might fast-track the treatment.

Despite HCPs’ proposing that the patients invite family members to come to the hospital, patients generally insisted on returning home. The participants then tended to give the patients a deadline for returning and they warned them that traditional healing might not necessarily be effective. They reminded the patients that the ultimate decision was theirs, and they ensured that the patients were well-informed about their condition. These strategies denote a paternalistic, individualistic approach and demonstrate limitations regarding veritable understanding of the patients’ cultural paradigms. Patients who have cultural health beliefs, embedded in traditional healing, believe in the curative capacity of traditional medicine (Muhamad et al. 2012).

Similarly, reinforcing individualism regarding decision-making negates the collectivistic paradigm within which Zulu patients operate (Washington 2010). Although HCPs might be well-intentioned and focused on life-saving, strategies used for patients who insist on going home are not indigenously collaborative and, consequently, they favour Western approaches to medical decision-making. The HCPs in this study demonstrated a willingness to engage collaboratively and cross-culturally. An awareness of the use of paternalistic strategies in response to patients who insist on going home would therefore assist HCPs to change this approach.

### Limitations of the study

Although themes were repeated within the focus groups, all the members of the MDT were not available for the data collection and this may have limited our ability to achieve data saturation. Furthermore, our use of discipline with specific focus groups may have limited our opportunity to assess the inter-professional functioning of MDT members. We also note that the use of other qualitative data-gathering techniques may have complemented the focus groups and enhanced our data.

## Conclusion

This study, the first of its kind in the South African context, explored the cultural factors associated with discussing treatment options and identified strategies for responding to these factors. The cultural factors highlighted included patients’ beliefs about amputation, their need to access traditional healing and their requirements regarding collective treatment decision-making. The participants highlighted the importance of balancing respect for patients’ cultural preferences with the need to expedite treatment decision-making in order to improve the prognostic outcomes. Their proposed strategies for responding to patients’ aversive responses to amputation, entailed timing the treatment discussion, using patient models and visual media to ease patients’ anxieties, referring patients appropriately and mobilising support.

Strategies for attending to cultural and health beliefs impacting treatment decision-making included initiating the cultural discussion, seeking and demonstrating an understanding of patients’ cultural beliefs, liaising directly with the family and cultural decision-makers, learning about the patient’s culture and working collaboratively with traditional healers. While the participants reported significant efforts to provide culturally competent care, medical paternalism emerged in response to patients who insisted on going home to engage in their traditions.

The strategies that we present will be useful in other cross-clinical settings where patients belong to collectivistic cultures and observe traditions and other practices that are significantly different from Western medical approaches. We recommend that guidelines for culturally competent communication with cancer patients should explicitly discuss the issues of managing culturally discordant treatment options. This study will contribute to the development of a guideline that would achieve this goal.
